# Leveraging sequence-to-sequence models for semantic annotation of Dutch pathology reports

**DOI:** 10.1016/j.jpi.2025.100534

**Published:** 2025-12-05

**Authors:** M. Siepel, G.T.N. Burger, Q.J.M. Voorham, R. Cornet, I. Calixto, I. Vagliano

**Affiliations:** a Amsterdam UMC, University of Amsterdam, Department of Medical Microbiology and Infection Prevention, Amsterdam Public Health Research Institute, Digital Health & Methodology, Amsterdam, the Netherlands; b Amsterdam UMC, University of Amsterdam, Department of Internal Medicine, Amsterdam Public Health Research Institute, Digital Health & Methodology, Amsterdam, the Netherlands; c Amsterdam UMC, University of Amsterdam, Department of Medical Informatics, Amsterdam Public Health Research Institute, Quality of Care & Methodology, Amsterdam, the Netherlands; d Symbiant Pathology Expert Centre, Hoorn, the Netherlands; e Palga Foundation, Houten, the Netherlands; f Amsterdam UMC, University of Amsterdam, Department of Medical Informatics, Amsterdam Public Health Research Institute, Digital Health & Methodology, Amsterdam, the Netherlands; g Amsterdam UMC, University of Amsterdam, Department of Medical Informatics, Amsterdam Public Health Research Institute, Mental Health & Methodology, Amsterdam, the Netherlands

**Keywords:** Pathology, Semantic annotation, Deep learning, Transformer, T5, Autoregressive entity retrieval

## Abstract

Palga Foundation is responsible for indexing Dutch pathology data across the Netherlands, which relies on annotations of pathology reports. These annotations, derived from the conclusion text, consist of codes from the Palga thesaurus, serving patient care and scientific research. However, manual annotation by pathologists is both labor-intensive and prone to errors. Therefore, in this study, we seek to leverage sequence-to-sequence transformer models, particularly Text-To-Text Transfer Transformer (T5)-based models, to generate these annotations. Additionally, we investigate a constrained decoding (CD) approach that encodes domain knowledge. We compare a standard multilingual T5 model (mT5) with our own T5 model (PaTh5.NL) pre-trained using Palga data with the goal of better aligning the model's learned representations with the specific structure, terminology, and annotation conventions used in Dutch pathology reports. We fine-tune both pre-trained models using default (DD) and CD and compare both decoding strategies. Performance is assessed using Bilingual Evaluation Understudy (BLEU) scores for quantitative evaluation and case-based evaluations for qualitative assessment, where we use the generated codes to retrieve patients from the Palga database. Quantitative evaluations indicated that our two fine-tuned PaTh5.NL models significantly outperformed the fine-tuned mT5 model, particularly for shorter histology and cytology reports, but performance of all models declined on longer or complex reports. The case-based evaluation revealed that, despite higher BLEU scores, the PaTh5.NL models did not consistently outperform the mT5 model in retrieving relevant patients. This study demonstrates that fine-tuned T5-based models can enhance the annotation process for Dutch pathology reports, though challenges remain regarding complex conclusion texts, especially in histology and autopsy reports. Future research should focus on expanding gold-standard datasets and developing post-processing algorithms to improve annotations' generalization.

## Highlights

• Fine-tuned T5 transformer models can automatically annotate Dutch pathology reports.
• Quality of the generated annotation is good for reports with short conclusions.
• However, the quality decreases with report conclusion complexity.
• Further refinement of the model scan be beneficiary for the annotation quality.

## Graphical abstract

**Figure:**
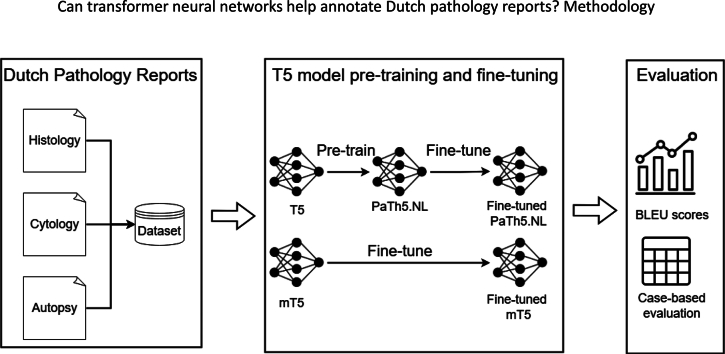

## Introduction

**Fig. 1:**
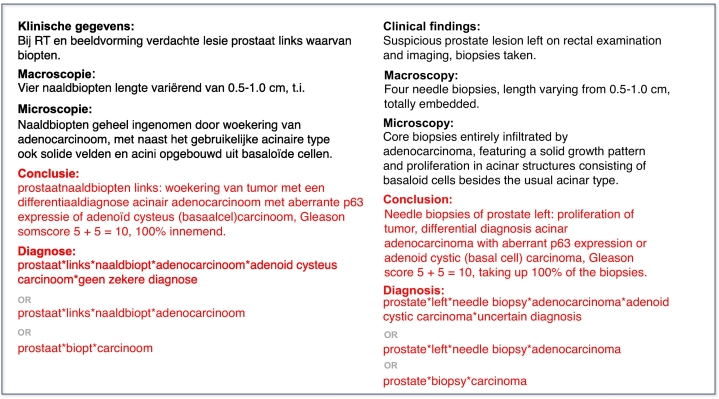
Example Dutch pathology report. Report sections used in this article are in red. The original Dutch text is shown on the left and the English translation on the right. (For interpretation of the references to color in this figure legend, the reader is referred to the web version of this article.)

Accurate and efficient annotation of pathology reports is crucial for the effective indexing and retrieval of pathology data. In the Netherlands, the Palga Foundation^3^ plays a pivotal role in this process, collecting and indexing nationwide pathology data to facilitate patient care and scientific research.^1^^,^^2^ To facilitate this indexing process, all pathologists in the Netherlands are required to annotate each pathology report using a set of Palga codes, which are based on SNOMED II codes and constitute the Palga thesaurus.^3^ Pathologists use these codes to summarize the conclusions of pathology reports into combinations of codes, forming one or more *code series*, as exemplified in Fig. 1 and Table 1. As indicated in Fig. 1, there are several allowed ways to annotate a report. Effective annotations lead to correct indexing, which in turn allows for the retrieval of appropriate data for from the Palga-database.

**Table 1:**
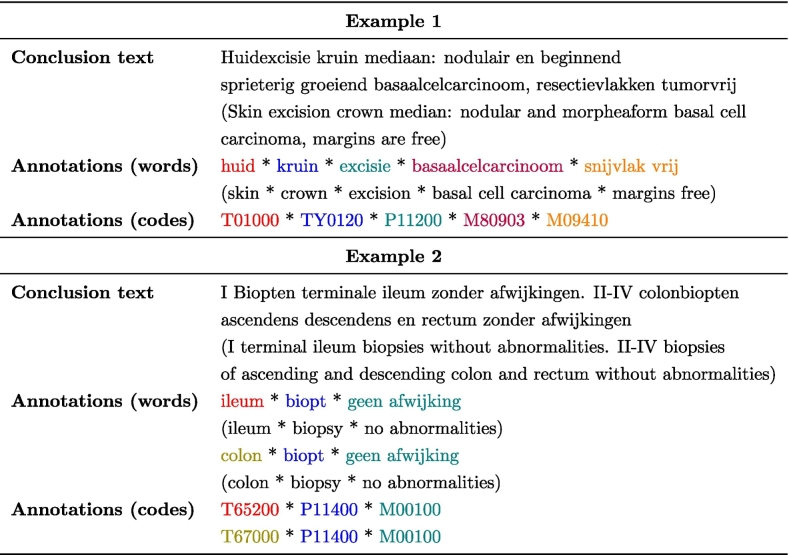
Example conclusion texts and corresponding Palga annotations. The second example contains two code series for one conclusion. Terms and codes are separated by an asterisk. A new line indicates the start of a new code series. Annotation words and their corresponding codes are colored accordingly. Translations between brackets.

The task of manually annotating these reports is both time-consuming and error-prone. To address this, two tools are currently available. The first tool is the Palga Protocol Module for synoptic reporting. Here, the reporting pathologist fills out a form, and the tool automatically generates the Palga code annotations in the background.^4^ This tool, however, is only available for frequently occurring (oncology) specimens. The second tool is the Pathology Report Annotation Module (PRAM), a rule-based tool currently deployed in several pathology labs in the Netherlands.^5^ PRAM generates annotations based on the report conclusion text through pre-processing steps followed by heuristic rules. Whereas it performs adequately on short sentences, it struggles with longer conclusion texts. Also, PRAM requires pathology terms in the conclusion text to have an exact match in its rules to retrieve codes, which makes it prone to mistakes due to wrong or alternative spellings or words. For example, in the example report in Fig. 1, PRAM would generate prostaat*links*adenoid*naaldbiopt*acinair adenocarcinoom*carcinoom, misinterpreting adenoid as a topographical descriptor rather than part of the correct diagnosis, adenoid cysteus carcinoom. This misinterpretation occurs due to the prefix (basaalcelcarcinoom) preceding carcinoom in the conclusion, which disrupts the recognition of the correct diagnosis. As a result, incorrect annotations may be generated, leading to sub-optimal data retrieval from the database. Furthermore, it is important to note that, apart from the rule requiring annotation sequences to include a topography, procedure, and morphology code (in that order), no universal annotation standard exists. Pathologists do not adhere to a single convention for annotating conclusions, meaning there is no absolute gold-standard for pathology report annotation. This variability is also shown in Fig. 1, where the diagnosis is annotated in three different yet technically correct ways, each differing in the level of detail. The field of natural language processing (NLP) provides potential solutions for annotating pathology reports and addressing the previously mentioned limitations. NLP is a research field part of artificial intelligence (AI) focusing on endowing computers with human language understanding and generation capabilities. Recently, Transformer-based^6^ large language models (LLMs) such as BERT,^7^ Text-To-Text Transfer Transformer (T5),^8^ and GPT-4^9^ have significantly progressed NLP, demonstrating a remarkable ability to interpret and organize complex textual data. These LLMs undergo extensive pre-training on large amounts of free-text data through self-supervised learning techniques, allowing them to learn general language patterns. When fine-tuned for specific tasks by modifying their weights to adapt to task-specific data, they raise the bar in performance on many NLP tasks, such as named entity recognition,^10^ sentiment analysis,^11^ machine translation,^12^ and question answering.^13^

Transformer models come in various forms. Encoder-only models like BERT receive text as inputs and compute embeddings for these inputs, which are useful in classification tasks.^14^ Decoder-only models like GPT-4 generate text word by word, which makes them useful in text generation tasks.^15^ However, sequence-to-sequence Transformer models,^16^^,^^17^ which combine encoder and decoder elements, are particularly promising for annotating pathology reports. These models process sequences of text as input and produce different sequences as output, leading them to achieve impressive results on various tasks such as summarization,^18^ translation,^19^ and question answering.^20^ Deploying a sequence-to-sequence model for annotating pathology reports could save significant time and improve the quality of indexed pathology data, enhancing Palga's ability to retrieve relevant data for research.

Various sequence-to-sequence models exist, such as T5, BART,^21^ PEGASUS,^22^ and MarianMT.^23^ T5 is a series of LLMs developed by Google and designed to frame every NLP task as a text-to-text problem, where tasks like classification, summarization, or translation are reformulated to take text as input and produce text as output. This includes, for example, converting a sentence in one language into its equivalent in another language, or generating a sequence of diagnostic codes from a clinical report. This approach allows T5 to handle a wide range of NLP tasks with a consistent format, making it particularly suitable for generating structured outputs, as all tasks are presented in a uniform text-to-text format regardless of their underlying complexity or type. Therefore, it is also suitable for the generation of Palga codes, where maintaining the sequence of codes is critical. Furthermore, T5-based models have been successfully applied in medical contexts, including reshaping radiology reports and extracting relations in medical texts.^24^^,^^25^ Lastly, the multilingual T5 model (mT5), pre-trained on 101 languages including Dutch, further enhances its applicability for annotating Dutch pathology reports.

Whereas neural networks like T5-based models are expected to learn to predict the same codes a pathologist would use to annotate a conclusion text, it is particularly important for Palga that the annotations meet specific requirements. These include ensuring the presence of at least one topography and one diagnosis code and maintaining the order of topography, procedure, and diagnosis (for details, please refer to “Background”). Additionally, not all code combinations make sense. For example, coding “wervel” (vertebra) and “dermatitis” (inflammation of the skin) together or using mutually exclusive codes like “maligniteit” (malignancy) and “geen maligniteit” (no malignancy) should not occur. To implement the annotation rules specified by Palga, neuro-symbolic models, which combine traditional rule-based systems with machine learning methods, can be employed.^26^ Neuro-symbolic models leverage structured information and rule-based reasoning, achieving state-of-the-art results in various tasks such as International Classification of Diseases coding prediction,^27^ as well as enhancing rule-based systems that are commonly used in Clinical Decision Support Systems.^28^ One potential application of neuro-symbolic models in the context of sequence-to-sequence models is to leverage constrained decoding (CD). By constraining which codes can be predicted in the outcome, the model can be forced to generate output that follows the rules determined by Palga regarding the presence, order, and internal consistency of codes.

Multiple variants of CD for transformer models in healthcare have been previously proposed, such as adding a dedicated attention head.^29^ We adapt the idea proposed for autoregressive entity retrieval^30^ for the purpose of predicting sequences of valid Palga codes. Autoregressive entity retrieval systems, which have previously been successfully implemented for entity linking (disambiguating the identity of entities found in text), document retrieval (finding relevant documents), and event extraction (identifying and categorizing events within text), are still unexplored in the context of annotating medical texts.^30^^,^^31^ Specifically, our implementation relies on generating codes only allowed for continuations based on historical annotation patterns, from which we removed patterns that did not follow the specific annotation rules, described in “Background”, to ensure that the model only generates codes that exist, are valid, and make clinical sense.

We developed and validated a method that automatically annotated Dutch pathology reports and investigated whether the quality of these annotations was at least as good as manual annotations by expert pathologists. This study investigated *how well fine-tuned T5-based models could automatically annotate Dutch pathology reports compared to existing rule-based annotations*. Additionally, we assessed the impact of in-domain pre-training and CD on annotation quality. Specifically, we examined pre-training the T5 model architecture from scratch to evaluate the effect of in-domain pre-training on performance and explored whether using CD with Palga domain-specific rules further improved the overall quality of the generated codes.

## Background

Dutch pathology reports consist of several mandatory sections: clinical findings, macroscopy, microscopy, conclusion, and annotations. Optional sections such as immunology and additional examination may also appear. The annotations can be viewed as a summary of the report conclusion section. Thus, the annotation process results in representing the report conclusion as a series of predefined codes from the Palga thesaurus, forming one or more *code series*.

A code series is a sequence of Palga thesaurus codes specifying, in this order, at least one topography (the site(s) the tissue came from), at least one procedure (e.g., biopsy, excision)—though in cytology reports, the procedure code may be omitted—and at least one diagnosis (morphology or related classification). After these mandatory codes, additional codes (including secondary topographies, for example, to indicate a metastatic process) may follow. Each pathology report must be annotated with at least one code series. If the conclusion text contains multiple diagnoses or involves multiple topographies, multiple code series may be used; this is often done when multiple topographies are involved, but it is not strictly required. For examples, see Fig. 1 and Table 1.

The codes in the Palga thesaurus are divided into three categories: topography (T), procedure (P), and diagnosis (M = morphology, plus D = disease, E = etiology, and F = function). Each code begins with one of these letters, followed by capitals and digits; for instance, TYY980 (right), P11200 (excision), or M80903 (basal cell carcinoma). Code combinations such as T96000P11100 (thyroidectomy)—composed of valid single codes T96000 (thyroid) and P11100 (resection)—are also possible.

Table 1 shows two examples of conclusions and their code series. When a report is submitted to Palga, each code series is checked to ensure it meets the minimal requirements (i.e., at least one topography, one procedure when required, and one diagnosis). This process guarantees the presence of a correct code series in the report when it is incorporated into the Palga database.

In clinical practice, a pathologist dictates the report using a speech-recognition program, finishing with the conclusion, and will thereafter add one or more code series. Alternatively, code series can be generated by the Palga Protocol Module in cases for which synoptic reporting is available, or PRAM can be used. The advantages of this are relieving the pathologist from the annotating duty and having annotations that are uniform for any given conclusion text. However, the Palga Protocol Module is only available for a limited set of (oncology) cases, and the performance of PRAM depends on the length and quality of the conclusion text. Consequently, when these automated tools do not yield adequate results, pathologists must resort to manual annotation. However, due to heavy workloads and time constraints, these annotations could remain terse, inconsistent, and less effective. As a result, the quality of manually added codes can be highly variable and not as comprehensive as desired.

## Methods

### Data

All pathology reports used in this research were supplied by Palga. The histology and cytology reports were collected between 2013 and 2023. Autopsy reports, which are less frequent, were collected over a broader timeframe from 2000 to 2023 to ensure a sufficient amount of training data. No distinction was made regarding the report source (pathology department, authoring pathologist). Reports with annotations created by PRAM or Palga Protocol Module were excluded as well as reports of cervical cytology specimens.^4^ No patient-identifying information was included. Each report was categorized into one of three types: histology (tissue examination, T), cytology (examination of cells, C), or autopsy (Dutch word for autopsy, “sectie”, S). Additionally, each report contained the year the report was received, the report conclusion text, and the annotations in the form of one or more code series, in textual form (term words), and in the corresponding Palga code form.

For the purpose of training and testing our model, the following four datasets, which are also summarized in Table 2, were provided by Palga.

**Table 2:** Data statistics for the pre-trained, fine-tuned, gold-standard, and case-based datasets. For each dataset and report type, the number of samples, the median length with interquartile range (IQR), and the median number of codes with IQR are given.

	No.	Median length (IQR)	Median nr. of codes (IQR)
*Pre-train*			
Histology	878.611	15 (9–26)	4 (3–5)
Cytology	118.082	11 (7–17)	4 (3–5)
Autopsy	3.307	124 (65–207)	4 (3–8)

*Fine-tune*			
Histology	145.100	16 (10–28)	4 (3–5)
Cytology	46.605	12 (8–18)	4 (3–5)
Autopsy	55.670	57 (23–116)	6 (3−11)

*Gold-standard*			
Histology	996	19 (11–34)	6 (5–8)
Cytology	300	10 (6–15)	4 (4–5)
Autopsy	200	128 (61–223)	15 (9–21)

*Case-based*			
Histology	158.846	17 (9–34)	4.0 (3–6)
Cytology	57.998	10 (7–18)	4.0 (3–5)
Autopsy	808	87 (49–163)	10.0 (5–16)

We used Palga data for both pre-training and fine-tuning our models. The process of pre-training involved self-supervised learning on a large set of unannotated pathology conclusion texts. Specifically, the model learned language patterns by predicting masked words within these texts, thereby acquiring general domain-specific knowledge. The process of fine-tuning then adapted this general knowledge to our specific annotation task, using smaller datasets with annotated examples. This stage involved supervised learning, where the model was trained to predict structured annotations from conclusion texts. Fine-tuning makes the model task-aware and sensitive to label-specific patterns.

1. The pre-train (PT) dataset included 878.611 histology, 118.082 cytology, and 3.307 autopsy conclusion texts, collected between 2013 and 2023. This dataset was used solely for pre-training. We randomly split it into three datasets: PT training (80%), PT validation (10%), and PT test (10%).
2. The fine-tune (FT) dataset included 145.100 histology, 46.605 cytology, and 55.670 autopsy conclusion texts, as well as the corresponding annotations provided by the pathologists. It was used to fine-tune, validate, and test our models. We randomly split it into three datasets: FT training (80%), FT validation (10%), and FT test (10%). We used temporal splits, i.e., the training data spanned 2000–2016, whereas the validation and test data covered 2017–2023, ensuring they reflected recent trends. This method accounted for data changes over time, acting as external validation.
3. The gold-standard (GT) test dataset included 996 histology, 500 cytology, and 200 autopsy conclusion texts with detailed annotations. The histology section of the GT test set originates from 2015, as it was created for a prior research project.^5^ The cytology and autopsy subsets were manually annotated for this study and were sampled from the FT train set, covering reports dated between 2000 and 2016. As such, the GT test set does not follow the temporal split protocol used for the FT test set. This means that, unlike the FT test set, the GT test set was not temporally separated from the training data. This dataset was created to better assess each model's detailed annotation capabilities, as the FT test dataset is expected to contain sub-optimal annotations from the original pathologists. This is supported by the higher average number of codes per annotation in the GT test dataset compared to the FT test set displayed in Table 2. This gold-standard dataset was used solely as test data and will be referred to as the GT test dataset from here on.
4. The case-based evaluation dataset included 158.846 histology, 57.998 cytology, and 808 autopsy conclusion texts. This dataset was used in our case-based evaluation, which is explained in “Qualitative case-based evaluation”.

### Model development

#### Models

We use two pre-trained models: the publicly available multilingual T5 model mT5, pre-trained for general multilingual understanding,^5^ and the PaTh5.NL model, which has the same architecture as the original T5 model, on which mT5 is also based, but which we pre-trained ourselves with the PT train dataset provided by Palga.

In total, we fine-tuned three models. Our first model is based on mT5 and is fine-tuned using the FT dataset with greedy decoding during training. For validation and testing, beam search decoding (a decoding algorithm that explores different sequences and selects the one with the highest probability)^32^ was used to calculate validation scores and select the best-performing model during training. Our second model is PaTh5.NL default decoding (DD), based on the pre-trained PaTh5.NL model, which followed the same training and evaluation procedure as the mT5-based model: greedy decoding during training and beam search for validation and testing. Our third model is PaTh5.NL CD, also based on PaTh5.NL, where we also trained using greedy decoding, but during validation and testing, we applied CD.

Both the mT5 and the T5-based models are available in different sizes, and we used the small version in our experiments. We also experimented with the base size, but it did not yield better results.

#### Pre-training

The PaTh5.NL weights were trained using the same architecture and training objective as the original T5 weights. The only difference between our implementation and the original was the custom tokenizer we created specifically for our task to ensure optimal alignment with our data. A tokenizer, which is responsible for breaking words and sentences into smaller units (tokens) that the model can process, plays a crucial role in how text is internally represented. These tokens can be whole words or smaller subword units. Our custom tokenizer resulted in a 65% reduction in tokens required to represent the reference annotations and a 46% reduction in tokens required to represent the input text compared to the default mT5 tokenizer. Details about the custom tokenizer and its performance are provided in Appendix A.

For pre-training, we used a batch size of 32 and a maximum length of 512 tokens. Input sequences longer than 512 tokens were split beforehand into chunks of 512 tokens and processed independently, so as not to lose any text. We pre-trained the model using the PT train for a total of 50 epochs and selected the best model based on the lowest loss on PT validation. Our pre-training encompassed all 800.000 conclusion texts in the PT train dataset, with an average length of 24 tokens, culminating in the model processing approximately 960 M tokens.

#### Fine-tuning

We used 10% of our fine-tune train dataset to perform a line search for multiple hyperparameters, as described in Table 3. All other hyperparameters, such as the model dimensions, were kept at their default values. We fine-tuned all models (mT5, PaTh5.NL-DD, and PaTh5.NL-CD) on FT train for 30 epochs and selected the best model based on the lowest validation loss on FT validation. The maximum input length was set at 512 tokens, which is also the maximum length that T5 models can process. For model fine-tuning, we selected the best-performing values for each hyperparameter.

**Table 3:** Parameter ranges for fine-tuning hyperparameters.

Hyperparameter	Search space	Best value
Training batch size	4, 8, 16, 32	8
Validation batch size	2, 4, 8, 16	4
Maximum output length	16, 32, 64, 128	128
Learning rate	1e-3, 5e-4, 1e-4, 1e-5	1e-4
Learning rate optimizer	AdamW, AdaFactor	AdaFactor
Learning rate scheduler	None, Slanted Triangular Learning Rate	None
Dropout rate	0.1, 0.2, 0.3, 0.4	0.1
Freezing all but X layers	0, 1, 3, 5	0

We used the default cross-entropy loss. We also experimented with a custom cross-entropy loss function aimed at improving the accuracy of predicting the correct number of code series in cases, where a conclusion report contained multiple code series. This loss function reweighted errors based on the difference between predicted and reference counts. As this approach did not yield consistent improvements during preliminary testing, we opted to use the standard cross-entropy loss for all experiments.

For our decoding technique, we used beam search with 6 beams, 2 beam groups, and a diversity penalty of 0.3.^33^ These settings were the result of a random search among various decoding strategies and parameter values. The strategies and ranges of values that we considered are shown in Appendix B.

#### Constrained decoding

For the model leveraging CD (PaTh5.NL-CD), we adapted the idea in the constrained autoregressive entity retrieval method of De Cao et al.^30^ for our problem. They leveraged a prefix tree (or Trie) *T* to ensure the generation of valid entries based on a dictionary. Specifically, they created a structure that allows their models to retrieve all valid continuations of entities after generating a partial entity, i.e., an *entity prefix*. For example, after generating “diabetes mellitus type”, valid continuations in the Palga thesaurus are “1” and “2”, and thus, the model is limited to only generating one of those continuations.

De Cao et al. used a knowledge base containing all valid entities to create their Trie. We cannot use such a knowledge base. Whereas it is known beforehand what entities to generate, namely any annotation in the dataset, we cannot predetermine which annotations have to be generated and in what order solely based on the dataset. To address this, we created our Directed Acyclic Graph (DAG) based on historical annotation data, using the entire ∼1 M records in our pre-training dataset PT. By leveraging these records, we constructed a DAG containing all valid annotation sequences that have been observed in past annotations.

A Trie enforces a strict tree structure, meaning each node can have only one parent. However, in our use case, restricting a code to a single predecessor is not reflecting actual annotation practices, where a code can follow multiple different entities. For example, the annotation links (left) can follow both huid (skin) and pleura (pleura), depending on the case. A Trie would require duplicating links under both parents, leading to unnecessary redundancy. Instead, we use a DAG, which allows nodes to have multiple parents while maintaining valid sequence constraints.

The DAG is created by first initializing a root node and then iteratively adding sequences where each subsequent annotation is added as a child of the preceding annotation if it follows the rules described in “Background”. After each morphology annotation, an end-of-sequence (EOS) token is also included as a valid next token. This token ensures that the model can terminate its generation, which happens only after producing an EOS token. This process continues until all annotations are added for all original sequences in the pre-training dataset PT.

**Fig. 2:**
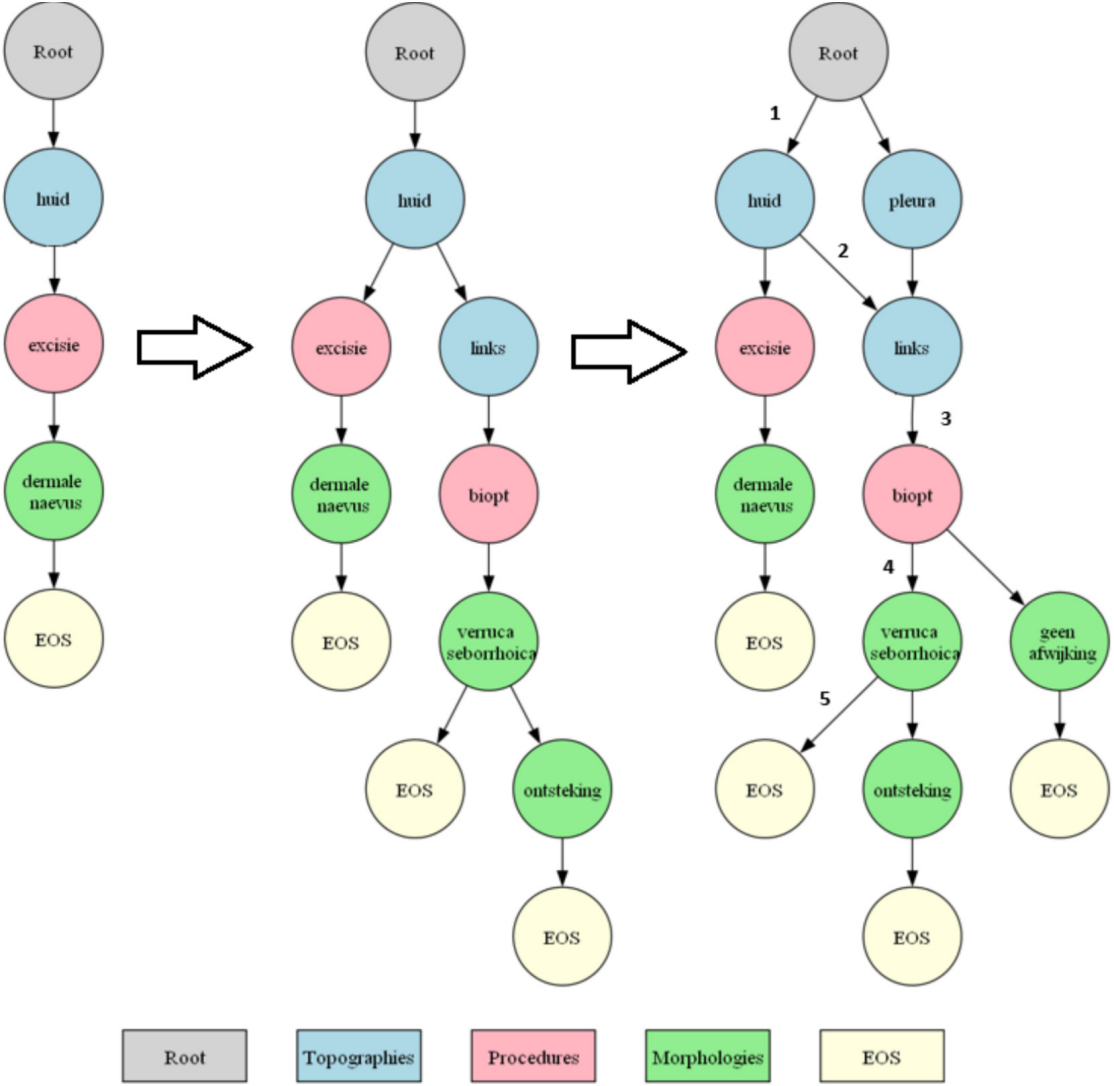
Example of a DAG construction in three stages. The leftmost DAG (stage 1) was constructed from a single sample annotation (huid*excisie*dermale naevus), the middle DAG (stage 2) was constructed by adding a second sample annotation (huid*links*biopt*verruca seborrhoica*ontsteking), and the rightmost DAG (stage 3) was constructed by adding a third sample annotation (pleura*links*biopt*geen afwijking). Translation: huid, skin; excisie, excision; biopt, biopsy; links, left; dermale naevus, dermal nevus; verruca seborrhoica, seborrhoic keratosis; ontsteking, inflammation; geen afwijkingen, no abnormality.

An example is shown in Fig. 2. In this example, a DAG is constructed using three annotation samples. The process of DAG construction is illustrated in three stages, showing the incremental addition of each sample. The leftmost DAG represents the structure after inserting the first sample (stage 1): huid*excisie*dermale naevus. The first layer after the root node consists of the annotation huid, which corresponds to the first entity allowed in any generated sequence. This is followed by excisie and subsequently dermale naevus. Because dermale naevus is a morphology annotation, an EOS token is added, marking the end of this sequence.

The middle DAG (stage 2) is an extension of the first, in-corporating the second sample: huid*links*biopt *verruca seborrhoica*ontsteking. The sequence starts with huid, which is already present in the DAG, and adds links as a new branch. Similarly, biopt is introduced as a child of links. Because this partial sequence continues beyond biopt, its child nodes verruca seborrhoica and subsequently ontsteking are added, ending with an EOS token.

The rightmost DAG (stage 3) includes the final sample: pleura*links*biopt*geen afwijking. The new annotation pleura is introduced at the first level, forming an alternative entry point. This is followed by links and biopt, both of which were already present due to the previous sequence. However, a new morphology annotation, geen afwijking, is added under biopt, requiring an additional EOS token at the end of the path. An example traversal of this DAG for generating a new sequence could be as follows. Starting at the root node, the model can initially generate huid or pleura. If huid is selected (step 1), the DAG returns a valid next option: links. Choosing links (step 2) leads to a single valid continuation: biopt (step 3). At this point, the DAG allows two valid continuations: verruca seborrhoica and geen afwijking. If verruca seborrhoica is chosen (step 4), the final annotation must be an EOS token (step 5), marking the end of the sequence.

This generation method has four benefits: (1) the model can only predict codes that have been annotated before, and therefore not hallucinate new codes that are not in the Palga thesaurus; (2) the model can only predict codes that are allowed to follow one another according to Palga's rules regarding code validity, thereby forcing the validity of the code series; (3) the model can only predict codes that in the past have followed one another, ensuring that the codes make clinical sense; and (4) the model can only predict an EOS token after generating a morphology, ensuring that each annotation includes at least one topography and one morphology. However, this comes at the cost of increased computational complexity, as the DAG must be traversed during generation to retrieve valid continuations.

### Model performance

To assess the performance for our models, we relied on quantitative performance measurements as well as qualitative case-based performance. For our quantitative evaluation, we calculated Bilingual Evaluation Understudy (BLEU)^34^ and F1-scores. In addition to BLEU, we computed word error rate (WER), character error rate (CER), match error rate (MER), word info lost (WIL), word info preserved (WIP), and Recall-Oriented Understudy for Gisting Evaluation (ROUGE) to assess annotation similarity.^35^, ^36^, ^37^ These metrics (available in Appendix F) showed near-perfect correlation with BLEU (|*r*| > 0.98), suggesting that BLEU effectively summarizes the variation captured by the others. For clarity, we therefore report BLEU as the primary metric.

### Quantitative evaluation

To investigate the quantitative performance of our models, we carried out three experiments.

#### Experiment 1: Model performance on the FT test dataset

We calculated and compared BLEU scores for each of the models on both the FT test dataset and the gold-standard GT dataset. Moreover, we perform a bootstrap analysis to test for statistically significant differences between models. More concretely, we bootstrapped our test dataset 1000 times to report confidence intervals of the BLEU scores to assess the statistical differences between each of the models and a reference model (mT5). Further details regarding the bootstrapping analysis are given in “Implementation details”.

#### Experiment 2: Model performance versus conclusion text's length

We expected that, because longer conclusion texts and autopsy conclusion texts generally require more codes and include codes that are less common (i.e., they appear relatively infrequently in the Palga database, see Appendix C), our models' performance would vary significantly across these different subgroups. To investigate this association between report length and performance, we divided the FT test dataset into groups of length: 0–80 characters, 81–200 characters, 201–400 characters, and longer than 400 characters. Each of these groups was further subdivided based on the code type (T, C, and S) determined by Palga. This resulted in 12 groups on which we assessed performance. The lengths in each group were determined based on the various length distribution of each of the examination types to ensure that each group contained a representative number of conclusion texts. We calculated BLEU scores for each group in both the FT test and GT test datasets, and performed the same bootstrapping analysis as we did in experiment 1. We then analyzed the trend of the BLEU scores on the FT test across different text lengths and types.

#### Experiment 3: Model performance versus code frequency

**Fig. 3:**
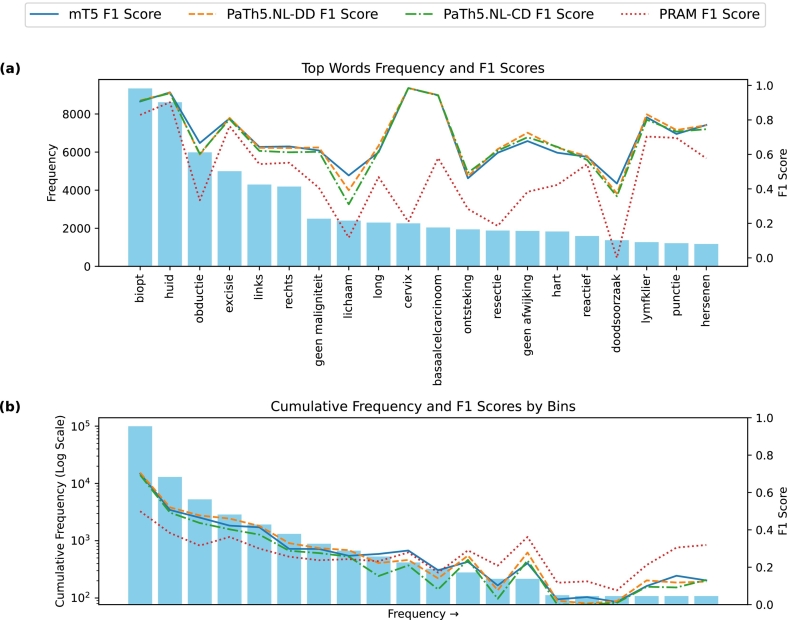
(a) F1-scores for the words associated to the 20 most frequent Palga codes. (b) Cumulative frequency (log scaled) and averaged F1-scores for 20 frequency-based binned groups of Palga codes.

We assessed the model's performance on frequently occurring codes versus rare ones to determine each model's performance on both common and uncommon codes. Because the Palga code usage distribution is heavily skewed (Fig. 3), it is important to investigate the performance on rare codes to uncover any bias the models might have towards more frequently occurring codes. To this end, we divided all the codes into 10 groups based on their cumulative frequency and calculated the average F1-score for each group. Additionally, we calculated the F1-score for each of the 20 most common codes. For these evaluations, we did not use BLEU scores because performance is measured at the individual code level. Instead, we computed precision and recall scores per-code, which are combined in the F1-score.

### Qualitative case-based evaluation

We also qualitatively assessed our models' performance in a case-based setting. Annotating pathology reports with Palga codes enables their retrieval for patient care and for scientific research. Pathologists and researchers submit (combinations of) Palga codes to Palga in order to find reports concerning patients with a specific diagnosis. Palga then executes a query on the nationwide pathology database to retrieve the desired report and/or patient population (Palga query). A single patient may have multiple related reports in the results.

As stated before, annotations added by the pathologist manually can be terse and inconsistent due to lack of a gold-standard for the creation of these annotations. Therefore, a reliable, automatic annotation system producing consistent Palga codes could be a valuable addition to, or replacement of manual annotations. We performed this qualitative case-based evaluation to get an impression of the contribution of automatic annotation to the number of retrieved reports and patients for a given Palga query.

To perform the evaluation, we obtained from the Netherlands Cancer Registry (NKR), maintained by the Netherlands Comprehensive Cancer Registry (IKNL)^6^ four anonymized collections of patients who were known to have a certain disease. We investigated sets of patients with the following diseases: neuroendocrine tumors, thyroid carcinomas, small intestine cancers, and colorectal carcinomas. These four malignancies were selected because they allowed reliable linkage with the NKR, varied in prevalence (from common, such as colorectal carcinoma, to rare, such as small intestine cancers), and, based on prior experience, differed in annotation consistency. Colorectal cancer is generally well-annotated, whereas others, such as neuroendocrine tumors, are expected to be more variable. In all cases, the selected queries could be formulated entirely using Palga codes.

For each patient who at one point suffered from one of the four diseases, all reports connected to these patients were retrieved from the Palga database. In total 218,310 reports were retrieved, further statistics regarding this dataset is provided in “Data”. Among these reports, some contained diagnoses related to the specific disease the patient had according to the NKR, but not all—several reports referred to other conditions the patient was diagnosed with during their lifetime. This yielded four sets of reports, each corresponding to one of the patient sets supplied by the NKR.

The reports from the Palga database already contained manual annotations by the original pathologist. To these, we added annotations generated by the models used in this article: mT5, PaTh5.NL-DD, PaTh5.NL-CD, and PRAM. For each disease type, a Palga query (using only the annotations, not the conclusion text) was designed and run by one of the authors (RV) using the original annotations and the annotations created by our four models. The Palga codes used in the queries are detailed in Appendix D. This procedure yielded results for each model for each disease, namely the number of patients correctly retrieved. A patient was considered retrieved by a model if the Palga query in question indicated a hit on at least one of the reports belonging to that patient. In this setting, false negatives correspond to patients who were not retrieved due to missing annotations, whereas true negatives represent patients with the disease but without relevant pathology reports in the Palga database.

For each report set, we counted: (1) the number of patients retrieved only by original codes—these patients were found using the original codes but were missed by our models; (2) the number of patients retrieved only by the model—these patients were identified by our models but would have been missed using only the original codes; (3) the number of patients retrieved by both original codes and the model (intersection)—these patients would have been found regardless of the method; and (4) the total number of patients retrieved by the model—this value was used to compare the retrieval performance of each model.

To understand why our models missed reports found using original annotations, we examined up to 100 records per query for each model. We categorized these reasons as follows: (1) Absence of relevant information: The report text contained no content on which annotations could be based. (2) Essential code not recognized: The model generated correct and relevant codes but omitted the specific code required for the query. (3) Incorrect codes: The model misinterpreted the report text and assigned incorrect codes. (4) No codes: Only PRAM could produce a “no codes” output when no valid code sequence aligning with Palga guidelines could be generated.

### Implementation details

All models were trained on a single NVIDIA A100 GPU with 40GB of RAM, provided by the Snellius, the Dutch National Supercomputer. Furthermore, all model loading, saving, and training were implemented using the HuggingFace library.^38^ Fig. 1 was created using the Python implementation of the Graphviz library, all other figures were created using the Matplotlib library in Python.^7^,^8^ To calculate the BLEU scores and carry out the bootstrapping analysis mentioned in Section “Quantitative evaluation”, we used the default implementation of the SacreBLEU package.^9^ We used Python version 3.11.^10^

### Permissions

Access to all pathology report datasets used in this study was granted by Palga. Approval to use patient data from the NKR was obtained from the Netherlands Comprehensive Cancer Organisation (IKNL).

## Results

### Quantitative evaluation

#### Experiment 1: Model performance on the FT test dataset

Table 4 presents the BLEU scores and confidence intervals for each model on the full FT test and GT test datasets, including *p*-values indicating statistical differences when comparing each model to the reference model (mT5). On both the FT test and the GT test dataset, PaTh5.NL-DD outperforms the other models.

**Table 4:** BLEU score and *p*-values for mT5, PaTh5.NL-DD, PaTh5.NL-CD, and PRAM on the FT test and gold-standard test datasets, with the mT5 model serving as the reference. “+” denotes a statistically significant BLEU increase and “−” a decrease. The *p*-value is listed as N/A for the mT5 model because it was used as the reference for comparison. Best performance is shown in bold.

	Test dataset		Gold-standard dataset	
Model	BLEU ± CI	*p*-value	BLEU ± CI	*p*-value
mT5	30.5 ± 0.5	N/A	18.4 ± 1.5	N/A
PaTh5.NL-DD	**34.6** **±** **0.6+**	<0.001	**28.1** **±** **1.8+**	<0.001
PaTh5.NL-CD	33.5 ± 0.5+	<0.001	26.3 ± 1.7+	<0.001
PRAM	9.9 ± 0.2-	<0.001	22.9 ± 1.5+	<0.001

#### Experiment 2: Model performance versus conclusion text's length

Table 5 shows the BLEU scores and confidence intervals for each model across different subgroups, with the best-performing model for each subgroup highlighted in bold. “+” indicates a statistically significant improvement in the BLEU score compared to the mT5 reference, whereas “−” indicates a statistically significant decrease.

**Table 5:** BLEU scores for the mT5, PaTh5.NL-DD, PaTh5.NL-CD, and PRAM across subgroups differing in size (in characters) and type. “+” denotes a statistically significant BLEU increase compared to mT5, whereas “−” denotes a decrease. Statistical significance is not provided for the mT5 model, as it serves as the reference for comparison. Best performance per dataset is shown in bold.

Length	Count	mT5	PaTh5.NL-DD	PaTh5.NL-CD	PRAM
*Test dataset*					
Histology					
0–80	3440	43.5 ± 1.6	**46.0 ± 1.6**^**+**^	45.0 ± 1.6^+^	29.5 ± 1.3^−^
81–200	7946	43.0 ± 0.9	**45.9 ± 1.0**^**+**^	45.1 ± 1.1^+^	20.4 ± 0.7^−^
201–400	3722	33.0 ± 1.1	**42.6 ± 1.3**^**+**^	41.1 ± 1.3^+^	15.6 ± 0.8^−^
400+	1277	15.7 ± 2.8	28.3 ± 3.4^+^	**29.2 ± 3.5+**	6.9 ± 2.4^−^
Cytology					
0–80	1892	48.6 ± 3.4	**50.4 ± 3.3**	48.3 ± 3.4	19.5 ± 2.2^−^
81–200	1834	57.0 ± 2.0	**57.2 ± 2.0**	55.1 ± 2.0-	16.8 ± 1.6^−^
201–400	409	34.6 ± 3.0	**43.1 ± 3.6**^**+**^	37.8 ± 3.7^+^	18.0 ± 2.5^−^
400+	388	15.7 ± 2.8	28.3 ± 3.4^+^	**29.2 ± 3.5**^**+**^	6.9 ± 2.4^−^
Autopsies					
0–80	112	14.5 ± 6.8	12.1 ± 6.5	**16.2 ± 9.6**	1.1 ± 0.5^−^
81–200	743	12.9 ± 2.0	15.9 ± 2.5^+^	**18.3 ± 2.5**^**+**^	3.7 ± 1.1^−^
201–400	1466	13.0 ± 1.3	**18.0 ± 1.4**^**+**^	16.7 ± 1.6^+^	5.4 ± 0.6^−^
400+	3856	11.5 ± 0.6	**18.7 ± 0.9**^**+**^	18.1 ± 0.7^+^	4.7 ± 0.2^−^

*Gold-standard dataset*					
Histology					
0–80	210	**47.0 ± 5.8**	46.8 ± 5.5	46.4 ± 5.7	42.1 ± 5.3^−^
81–200	432	28.0 ± 3.3	**39.5 ± 3.8**^**+**^	35.9 ± 4.0^+^	37.8 ± 3.5^+^
201–400	229	16.2 ± 3.1	27.8 ± 4.4^+^	24.9 ± 4.0^+^	**34.4 ± 3.6**^**+**^
400+	125	7.7 ± 2.5	14.9 ± 3.3^+^	16.2 ± 3.8^+^	**24.4 ± 2.7**^**+**^
Cytology					
0–80	240	39.4 ± 5.2	**42.1 ± 6.4**	38.4 ± 5.5	38.6 ± 5.9
81–200	219	**41.7 ± 4.8**	41.4 ± 5.2	37.7 ± 4.8^−^	28.5 ± 4.3^−^
201–400	35	30.5 ± 7.4	**42.2 ± 11.5**^**+**^	39.0 ± 10.4	21.5 ± 8.0^−^
400+	6	39.1 ± 19.1	42.7 ± 12.0	**45.1 ± 23.1**	14.7 ± 8.2^−^
Autopsies					
81–200	12	22.4 ± 16.7	**30.8 ± 22.3**	23.4 ± 14.4	8.5 ± 6.0
201–400	31	4.8 ± 3.2	11.5 ± 7.5^+^	**18.5 ± 8.8**^**+**^	16.1 ± 6.9^+^
400+	157	4.1 ± 1.3	**18.9 ± 3.1**^**+**^	16.0 ± 3.1^+^	12.9 ± 1.4^+^

On the FT test dataset subgroups, the PaTh5.NL-DD model is overall the best model. The PaTh5.NL-CD model achieved the highest BLEU scores on the longest cytology texts, the longest histology texts and relatively short autopsy texts.

The pattern of the PaTh5.NL-DD and PaTh5.NL-CD models consistently outperforming mT5, with the highest performances for short histology and cytology texts, are less pronounced in the GT test dataset, and the PaTh5.NL-DD model is not as clearly the model with the best overall performance. The PaTh5.NL-DD model again significantly scored best on the longest autopsy texts, medium-length cytology texts, and short-length histology texts. The PaTh5.NL-CD model performed best on medium-length autopsy texts. PRAM yielded the best results on medium to long histology texts, but was outperformed by the PaTh5.NL-DD and PaTh5.NL-CD models on the test dataset. Also, on the gold-standard set, the mT5, PaTh5.NL-DD and PaTh5.NL-CD models performed on par.

**Fig. 4:**
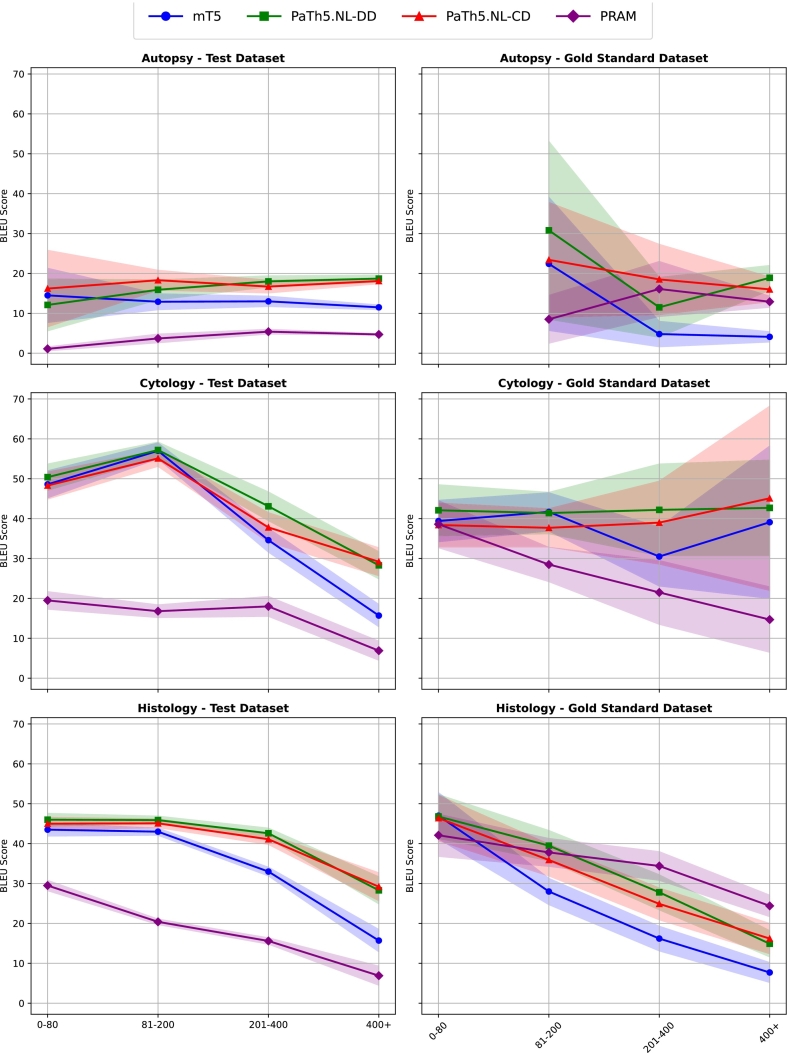
BLEU scores for test dataset and gold-standard dataset by type and length with confidence intervals shaded. Overlapping confidence intervals indicate no statistically significant differences between the models. No lines for autopsy—gold-standard dataset as there was no data in this subgroup. (For interpretation of the references to color in this figure legend, the reader is referred to the web version of this article.)

Fig. 4 also shows the trend per conclusion length and type. For histology and cytology conclusion texts, BLEU scores and conclusion length follow an inversely proportional trend, meaning longer conclusions have worse BLEU scores, and vice versa. For autopsy conclusions, longer reports have a slightly higher BLEU score on average. Significant differences between models are indicated by non-overlapping confidence intervals. This is the case for PRAM compared to the other models in all histology, cytology, and autopsy subgroups of the test dataset, where PRAM consistently has significantly lower performance. Additionally, PaTh5.NL-DD and PaTh5.NL-CD show significantly better performance for all medium-to-long conclusions (201–400, 400+) on the test dataset compared to mT5. In the gold-standard dataset, statistically significant differences occur less frequently and mainly highlight PRAM's significantly better performance for longer histology conclusions (201–400, 400+) and mT5's significantly worse performance compared to the other models on histology texts and the longest autopsy texts (400+).

#### Experiment 3: Model performance versus code frequency

Fig. 3a shows the F1-score of each model for the 20 most frequent codes in the test dataset. The most common code is “biopt” (biopsy), which appeared 9342 times. The second most common code is “huid” (skin), which appeared 8616 times. Then, in order, “obductie” (autopsy), “excisie” (excision), “links” (left), and “rechts” (right) appear 5985, 4996, and 4293 times. Together, these 5 codes make up 25.9% of all codes in the test dataset. The top 20 codes make up 49.2% of all codes, speaking to the skewness of the data. Within these 20 most common codes, the mT5, PaTh5.NL-DD, and PaTh5.NL-CD models perform similarly. Only PRAM performs worse on almost all common codes.

Fig. 3b shows averaged F1-scores of each model for binned cumulative frequency of Palga codes. Average F1-score generally decreases as frequency decreases. Notably, PRAM, which underperformed with frequent codes, starts outperforming fine-tuned T5-based models in lower-frequency bins. Importantly, average frequency of codes within each bin decreases significantly. Specifically, average occurrences per bin are 921, 120, 18, 4, and 1 for the first, second, fifth, tenth, and fifteenth bins, respectively. Thus, bins with lower average occurrences might be more susceptible to minor performance variations.

### Qualitative evaluation

Table 6 presents the results of the case-based evaluation for each queried entity. Here, we present an overview of the retrieved patient counts. The corresponding report counts are discussed in Appendix E. We introduce the set notation used: the total patient count retrieved by the model is denoted as *M*, and the total number of patients retrieved by the original codes as *O*. Patients retrieved only by the original codes are denoted as *OM*, patients retrieved only by the model as *M*\*O*, patients retrieved by both as *O*∩*M*.

**Table 6:** Case-based evaluation: results for four entities: neuroendocrine tumors, thyroid carcinomas, small intestine cancers, and colorectal carcinomas. For each model, we report the total patients retrieved by the original codes (*O*), by the model (*M*), the patients found only by the original codes (*O*\*M*), only by the model (*M*\*O*), and by both (*O*∩*M*).

Model type	*O*	*M*	*O*\*M*	*M*\*O*	*O*∩*M*
*Neuroendocrine tumors*					
mT5	2816	2559	446	189	2370
PaTh5.NL-DD	2816	2162	770	116	2046
PaTh5.NL-CD	2816	2124	835	143	1981
PRAM	2816	2501	523	208	2293

*Thyroid carcinomas*					
mT5	862	845	32	15	830
PaTh5.NL-DD	862	772	100	10	762
PaTh5.NL-CD	862	833	41	12	821
PRAM	862	854	21	13	841

*Small intestine cancers*					
mT5	2661	2553	154	46	2507
PaTh5.NL-DD	2661	2497	208	44	2453
PaTh5.NL-CD	2661	2521	182	42	2479
PRAM	2661	2651	88	78	2573

*Colorectal carcinomas*					
mT5	1484	1484	0	0	1484
PaTh5.NL-DD	1484	1484	0	0	1484
PaTh5.NL-CD	1484	1484	0	0	1484
PRAM	1484	1481	3	0	1481

For neuroendocrine tumors (*O*=2816), the mT5 model identifies the highest number of patients (*M* = 2559) while missing the fewest patients originally identified by the original codes (*O*\*M* = 446). PRAM identified the most patients missed by the original codes (*M*\*O* = 208) and identified the second-highest number of patients overall (*M* = 2501). The PaTh5.NL-DD model (*M* = 2162) and PaTh5.NL-CD model (*M* = 2124) identified the fewest total number of patients.

For thyroid carcinomas (*O* = 862), mT5 (*M* = 845) and PRAM (*M* = 854) again identified the most patients, whereas the PaTh5.NL-DD model identified the fewest patients (*M* = 772) and missed the most patients originally identified by the codes (*OM* = 100), compared to mT5 (*O*\*M* = 32), PaTh5.NL-CD (*OM* = 41), and PRAM (*O*\*M* = 21).

In the case of small intestine cancers (*O* = 2661), PRAM shows the best performance by identifying the most patients overall (*M* = 2651), the most new patients (*M*\*O* = 78), and missing the fewest patients originally identified by the codes (*O*\*M* = 88). Following PRAM, mT5 (*M* = 2553) identified the second-highest number of patients, with PaTh5.NL-DD (*M* = 2497) and PaTh5.NL-CD (*M* = 2521) trailing behind.

For colorectal carcinomas (*O* = 1484), all models retrieved nearly the same number of patients, with mT5, PaTh5.NL-DD, and PaTh5.NL-CD each retrieving 1484 patients, whereas PRAM retrieved slightly fewer at 1481 patients. Notably, none of the models contributed additional patients beyond those already identified by the original codes (*M*\*O* = 0). However, PRAM missed three patients that were retrieved by the other models (*O*\*M* = 3). The intersection (*O*\*M*) between the original codes and the models was nearly identical across all models, except for PRAM, which had a slightly lower overlap.

Overall, mT5 and PRAM demonstrated the best patient retrieval performance, with mT5 capturing the most neuroendocrine tumor cases and PRAM excelling in thyroid carcinomas and small intestine cancers. The PaTh5.NL-DD and PaTh5.NL-CD models retrieved a similar number of patients but had the lowest retrieval rates.

Table 7 displays the results from the in-depth evaluation regarding the reason for missing a patient for each of the models. Generally, all the models missed patients for the same reasons the same number of times. The primary exception is PRAM, which, in the cases of neuroendocrine tumors and thyroid carcinomas, less often misses a patient as a result of missing essential codes or generating the wrong codes. Also, PRAM is the only model that may generate no codes at all.

**Table 7:** Case-based evaluation: reason for missing results for four entities: neuroendocrine, thyroid, small intestine, and colorectal. For all cases, 100 patients were investigated, except thyroid carcinomas, for which only 76 patients were available.

Fault	mT5	PaTh5.NL-DD	PaTh5.NL-CD	PRAM
Neuroendocrine tumors, *N* = 100				
Absence of relevant information	34	34	34	34
Essential code not recognized	52	51	51	46
Wrong codes	14	15	15	11
No codes	–	–	–	9

Thyroid carcinomas, *N* = 76				
Absence of relevant information	23	23	23	23
Essential code not recognized	36	40	38	31
Wrong codes	17	13	15	8
No codes	–	–	–	14

Small intestine cancers, *N* = 100				
Absence of relevant information	78	78	78	78
Essential code not recognized	19	19	19	17
Wrong codes	3	3	3	2
No codes	–	–	–	3

Colorectal carcinomas, *N* = 100				
Absence of relevant information	88	88	88	88
Essential code not recognized	9	9	8	8
Wrong codes	3	3	4	3
No codes	–	–	–	1

## Discussion

### Main findings and interpretations

The aim of this study was to investigate how well fine-tuned T5-based models could automatically annotate Dutch pathology reports, in comparison to current rule-based or manually curated methods. We also examined whether in-domain pre-training and CD improved annotation quality. We compared our generated annotations to those in the Palga database, including the automatic annotations provided by PRAM, to determine whether they were of similar quality. Furthermore, we explored how pre-training custom T5-based models affected the performance of annotating Dutch pathology reports, and we studied whether Palga domain-specific rules could be enforced using CD to enhance the overall quality of the generated annotations.

#### Quantitative evaluation

Regarding how well fine-tuned T5-based models perform on annotations for Dutch pathology reports, BLEU scores higher than 30 were typically achieved on both the test and gold-standard datasets, which is commonly interpreted as indicative of good agreement with reference annotations.^39^ It is worthy to note that the BLEU score is a measure of token-level agreement between code series, i.e., how closely model-generated codes resemble those created by pathologists, but not a direct measure of annotation quality. Lower scores were obtained for medium-length histology texts (201–400) on the gold-standard dataset, the longest histology tests (400+), and all autopsy subgroups.

At the individual code level, the mT5 model and the PaTh5.NL-based models performed similarly, both generally achieving higher F1-scores than PRAM. However, the performance of all models, including PRAM, declined as the frequency of a code in the training data decreased. PRAM, in particular, struggled with specific codes such as “obductie” (autopsy), “lichaam” (body), and “doodsoorzaak” (cause of death). This likely stems from the fact that these terms are rarely explicitly stated by pathologists in the conclusion text, preventing PRAM from generating the corresponding codes. Nevertheless, these terms can often be inferred from context, which explains why the fine-tuned PaTh5.NL-based models and the mT5 model outperform PRAM in such cases.

#### Qualitative evaluation

The case-based evaluation revealed that the annotations generated by the mT5 model performed the most similar to the original codes for patient retrieval. PRAM found the most patients that were not retrieved by the original codes, potentially identifying patients that would otherwise have been missed. In all but the colorectal carcinoma case, all three fine-tuned T5-based models, and PRAM, identify fewer patients than they missed compared to the original codes, indicating a worse overall performance for patient retrieval. In the colorectal carcinoma case, the four models have no contribution to the number of patients retrieved. Based on our results, the fine-tuned mT5 model might be suitable to annotate specific cases (at least thyroid and colorectal carcinomas), but does not outperform PRAM, despite its higher BLEU scores.

Using machine learning models like ours for pathology report annotation presents a key challenge. The reference annotations provided by pathologists sometimes contain diagnoses that cannot be inferred from the conclusion text alone because the necessary information is missing. Our in-depth investigation of the reasons why patients were not retrieved by any of the generated codes revealed that in the case of small intestine cancers and colorectal carcinomas, 78 out of 100 and 88 out of 100 missed patients, respectively, were due to an absence of relevant information in the conclusion texts. The same issue occurred with neuroendocrine tumors, where 34 out of 100 missed patients could not be retrieved based on annotations because the required information was missing in the conclusion texts. For thyroid carcinomas, this happened in 23 out of 76 cases. In both situations, the models were unable to retrieve these patients based on annotations due to the absence of necessary details in the conclusion texts. This is an underlying issue in the data. Independent of the model used, these patients would not be correctly identified by automatic annotation methods. Addressing this limitation would require either improving the conclusion texts to include all clinically relevant information, or extending the models to process the full pathology report rather than relying solely on the conclusion. This could be a line of further study.

To better understand this issue, further investigation is needed to analyze both retrieved and missed patients, considering factors such as case rarity. Colorectal carcinomas, for example, are relatively common and well-represented in the training data, whereas small intestine cancers are much rarer, leading to fewer training examples. The frequency of related codes should also be taken into account, as our findings indicate a skewed distribution, with all fine-tuned T5-based models performing worse on less-frequent codes. This is particularly relevant for neuroendocrine tumors, which encompass multiple subtypes with diverse annotations, making each individual code rarer and potentially contributing to reduced model performance.

#### Pre-training benefits

Regarding how pre-training T5-based models affects the performance of report annotation, the PaTh5.NL-DD and PaTh5.NL-CD models achieve significantly higher BLEU scores on the full test set, the highest BLEU score in 9 out of 12 test set subgroups, and 4 out of 12 gold-standard subgroups in the quantitative evaluation. The PaTh5.NL-DD and PaTh5.NL-CD models perform similarly based on the frequency of the codes. In the case-based evaluation, the mT5 outperforms the PaTh5.NL-DD and PaTh5.NL-CD models on all but the colorectal carcinoma case, where the PaTh5.NL-DD and PaTh5.NL-CD models performs similar to the mT5 model.

To explain the increased BLEU scores (and thus, the ability to annotate more closely to the original annotations) compared to the mT5 model, we highlight two benefits of pre-training our own T5 model weights. First, because of its better initial alignment with the Dutch pathology language from the task at hand, the pre-trained weights were a better starting point for fine-tuning, which allowed the models to tune better to the fine-tuning data, resulting in higher BLEU scores. Second, pre-training a new model allowed us to train a custom tokenizer for this pre-trained model. Because a tokenizer is inherently connected to the pre-train weights, which means they must match for the model to work correctly, this was not possible for the mT5 model. This custom tokenizer allowed for more efficient tokenization. Because the text could be represented using fewer tokens, less context information was lost during tokenization. This means that the model retained more of the original meaning and nuances of the text. See also Appendix A for a more detailed explanation and Table A.1 for an example. Additionally, even though this did not necessarily result in higher BLEU scores, it was the custom tokenizer that allowed for the implementation of the CD algorithm. The advantages of using a custom tokenizer for pathology texts is in accordance with the findings of Santos et al.^40^

#### Constrained decoding

Concerning the effect of CD, we successfully forced our PaTh5.NL-CD model to generate annotations following the rules enforced by Palga by implementing a CD algorithm that implements an autoregressive entity retrieval system. The BLEU scores of the PaTh5.NL-CD model are statistically on par with those of the PaTh5.NL-DD model, albeit slightly lower. This can be explained by the fact that the reference annotations do not always follow the rules and thus, they cannot always be the same as the ones generated by our CD algorithm, resulting in slightly lower BLEU scores. Considering the qualitative evaluation, the PaTh5.NL-CD model did not outperform the PaTh5.NL-DD model regarding the number of patients retrieved. Also, according to the in-depth evaluation of reasons why patients were not retrieved, the PaTh5.NL-DD and PaTh5.NL-CD models perform similarly.

### Further interpretations

#### Conclusion text complexity

The (inverse) correlation between conclusion length, conclusion type, and BLEU scores can be explained by the fact that traditionally, most histology and especially cytology reports do not contain much text in the conclusion section because the results of the pathological examination are relatively straightforward. In case of complex cases, however, the pathologist will often add more information in the conclusion, thus creating a longer text. As this is the minority of the histology/cytology reports, our models have less reports of this kind to be trained on, resulting in worse performance on reports with long-winded conclusion texts. Also, conclusion texts of autopsy reports are not as straightforward and often long-winded. Often autopsy reports contain much clinical information or, on the other hand, are very terse. Therefore, autopsy reports with a long conclusion text may obscure the relevant information that needs to be annotated, and autopsy reports with a short conclusion text often do not contain all information required for adequate annotation.

In addition to text complexity, temporal variation in reporting language and annotation practices may also have influenced model performance. The fine-tune training set spanned 2000–2016, whereas the test data covers 2017–2023. The gold-standard dataset combines histology reports from 2015 with cytology and autopsy examples drawn from reports between 2000 and 2016. This mismatch means the model may not fully reflect newer terminology or evolving conventions, with malignant lymphomas being one relevant example of a domain where naming standards have shifted. The temporal split simulates real-world deployment not only by evaluating on temporally held-out data, but also by capturing actual performance degradation over time. This degradation highlights the need to address temporal drift. Implementing such models in pathology practice while maintaining model quality in the face of temporal drift would require periodic retraining. Approaches such as continual learning, discussed further in “Future work”, should be explored to mitigate the need for frequent retraining.^41^^,^^42^

#### BLEU score and case-based performance discrepancy

Interestingly, the PaTh5.NL-DD and PaTh5.NL-CD model achieved higher BLEU score than mT5 and PRAM. This means that the annotations generated by the PaTh5.NL-DD and PaTh5.NL-CD models are more similar to the original codes than the ones generated by the mT5 model. However, the PaTh5.NL-DD and PaTh5.NL-CD model performed generally worse than mT5 and PRAM in the case-base evaluation. This supports our view that higher BLEU scores reflect closer resemblance to the original codes but do not necessarily guarantee better annotation quality or improved patient retrieval outcomes.

We consider two reasons for these results. First, the PaTh5.NL-DD and PaTh5.NL-CD models attempt to annotate in more detail. Instead of annotating the code for a tumor of neuroendocrine origin (which was queried for), they attempt to annotate specific types of neuroendocrine tumors, such as “Merkel cell carcinoma” or “small cell carcinoma”. Although often correct, as reflected by the high number of “essential code not recognized” cases in Table 7, these annotations did not match the specific code patterns defined in our Palga queries,^11^ resulting in missed patients. Because the difference between a retrieved patient using general codes and a missed patient using specific codes is often only a single code, the BLEU score is not influenced, explaining how these models can still boast the highest BLEU scores. The second reason lies in the relatively worse performance of the PaTh5.NL-DD and PaTh5.NL-CD models on the gold-standard set compared to the test set. Whereas on the test set, the PaTh5.NL-CD and PaTh5.NL-DD models were the best-performing models, this does not hold for the gold-standard set. Here, the PaTh5.NL-DD and PaTh5.NL-CD models yielded lower BLEU scores than mT5 and PRAM for about half the subgroups, indicating potential overfitting to the sub-optimal fine-tuning data. Conversely, the mT5 model, which has lower BLEU scores, may have learned to annotate with better codes that differ from the sub-optimal ones in the test set, leading to lower BLEU scores. These better codes have resulted in the superior performance of mT5 in the case-based setting in comparison with the PaTh5.NL-DD and PaTh5.NL-CD models.

### Related work

Transformer-based models have significantly advanced the processing of medical texts, including pathology reports. PathologyBERT^40^ was pre-trained on histopathology reports and improved entity recognition and cancer classification tasks. Path-BigBird^43^ leveraged a long-document transformer architecture to effectively classify oncology-related attributes from extensive pathology report datasets. BioBERT^44^ and ClinicalBERT^45^ have demonstrated that domain-specific pre-training on biomedical and clinical texts enhances performance on healthcare-related NLP tasks. Similarly, a NLP framework utilizing FastText, a linear classifier-based text representation model, was applied to classify free-text cervical biopsy diagnoses into predefined categories, achieving high concordance with human annotations.^46^ Whereas these models excel in classification and entity extraction, our work differs by adopting a sequence-to-sequence approach for pathology annotation, offering structured outputs across a broad range of pathology subfields through the extensive Palga thesaurus.

CD ensures domain-specific rules are followed during text generation. Alignment-Enhanced Transformer was introduced to improve neural machine translation by integrating a dedicated attention head that captures external alignment supervision, ensuring that pre-specified translations are reliably incorporated into generated text.^29^ The Generative Entity Retrieval model employs an autoregressive approach for entity retrieval, using a constrained beam search with a prefix tree (Trie) to restrict generated outputs to a predefined set of valid entities, reducing memory footprint while maintaining high retrieval accuracy.^30^ Another framework for constrained text generation, designed to ensure compliance with structured data requirements in task-oriented dialog, introduces structured semantic representations and CD strategies to improve the semantic correctness of generated responses.^47^ To our knowledge, we are among the first to implement a DAG-based CD algorithm in healthcare, specifically for pathology annotation. Our approach ensures adherence to domain-specific coding standards without altering the underlying model architecture. Whereas previous CD methods have demonstrated improvements in translation and entity retrieval, our findings indicate that, in pathology annotation, CD primarily enhances adherence to clinical annotation standards, thereby ensuring greater consistency and validity in generated annotations.

### Strengths and limitations

This study has three primary strengths. First, we pre-trained a Dutch pathology-specific T5 model and introduced a novel CD method tailored to pathology report annotation. To the best of our knowledge, no prior research has presented either of these contributions. Specifically, we are the first to adapt an autoregressive entity retrieval approach for CD in a healthcare annotation context. Rather than relying on a predefined knowledge base, our method inductively learns valid annotation sequences from historical examples, enabling greater flexibility in reflecting real-world annotation patterns. To enforce correctness while preserving this flexibility, we use a DAG derived from past annotations. The DAG ensures that generated annotations are clinically valid and consistent with established usage. We believe these contributions represent meaningful progress in the semantic annotation of Dutch pathology reports. Second, we combined a quantitative and qualitative evaluation method. By measuring a quantitative metric like BLEU, we were able to determine the performance of all our models on the overall task of annotating pathology reports, independent of specific outcomes or diseases. This allowed us to calculate performance differences based on characteristics like the length and type of report. Additionally, by investigating the qualitative, case-based performance of each model, we were able to evaluate how well our models would perform if implemented by Palga. These insights are highly valuable and show that quantitative BLEU scores, while valid as a measure of agreement between codes created by pathologists and those generated by our models, do not fully capture all aspects of annotation quality or clinical utility. Third, because of the extensive data collection carried out by Palga Foundation, we were able to use a uniquely large amount of labeled data for pre-training and fine-tuning our models. The fact that the data were labeled, spanned multiple years, and categorized based on type allowed us to gain a unique insight into Dutch pathology data. This also allowed us to validate and test on more recent data, accounting for any temporal changes in the data, as an additional external validation. Furthermore, the approach of implementing and testing transformer networks, as described here, can serve as a useful framework for automatic annotation systems in situations where a sufficiently large dataset with manually annotated pathology (or other medical) texts is available or can be created. Because this method does not rely on a specific language or annotation system, it has broad applicability.

We have identified three primary limitations in this study. The first limitation is the sub-optimality of the provided annotations, as mentioned in the problem definition: annotations are often only partially complete or correct, either lacking relevant codes or containing incorrect codes. This has two main implications. First, during fine-tuning, models are fed reference annotations that are often not fully complete and correct. As a result, the model may also learn to predict incomplete or incorrect codes. Second, when calculating BLEU scores, predictions improving the original incomplete or incorrect reference annotations are scored as wrong, resulting in a BLEU score that potentially does not represent the actual completeness and correctness of the predictions. Concretely, lower BLEU scores could be the result of a model that predicts better codes than the original reference annotations. This may affect PRAM more than the other models because it uses fixed rules instead of learning from data. Unlike transformer models, it cannot adapt to noisy or incomplete annotations during training. As a result, it might generate more accurate or complete outputs than the references, but still receive lower BLEU scores for not matching them exactly.

The second limitation is the lack of a gold-standard for annotating Dutch pathology reports. Consequently, a large gold-standard dataset does not exist. In this study, we used a small, partially evaluated dataset, the annotations of which we consider to constitute a possible gold-standard. This dataset's cytology and autopsy sections were evaluated by only one pathologist (GB), who was involved in the research. The histology set was annotated and verified by four pathologists. Therefore, these datasets cannot be said to constitute an acknowledged gold-standard. Due to the dataset's small size, the results are susceptible to variability and may not be representative of certain subgroups. For instance, there are no samples for autopsy report texts of lengths 0–80, only 12 samples for lengths 81–200, and just 6 samples for cytology texts of 400+ characters lengths. This small sample size makes the results prone to significant changes when additional samples are added.

The third limitation is the lack of quantitative outcomes for our case-based evaluation. Because the dataset consisted exclusively of reports from NKR patients who were confirmed to have one of the queried diseases, every retrieved patient was a true positive, meaning false positives were not possible. The number of true negatives is likely low, as most oncology patients have at least one relevant pathology report recorded. Consequently, traditional evaluation metrics such as accuracy, precision, and F1 could not be meaningfully applied. In absence of these metrics, we considered calculating recall not meaningful and refrained from calculating this. Additionally, the in-depth evaluation of missed patients was conducted by a single pathologist. Ideally, this assessment should involve multiple pathologists to account for inter-rater agreement and reduce any subjectivity.

### Future work

We identified several lines of future work. First, a larger, completely verified gold-standard dataset should be annotated for the purpose of evaluating our models. Such a dataset would allow for a more reliable evaluation of the models' performance in achieving perfect annotations, which is crucial as the current datasets contain sub-optimal annotations. However, consensus on optimal annotation has to be reached first.

Second, to verify our results and further evaluate the case-based performance of our models, additional in-depth research is required into the matched and missed patients for each of our four cases. Whereas we investigated up to 100 patients per case that were not found by any of the models, we did not investigate the patients that were uniquely identified by each model and not by the original annotations. Understanding whether the patients uniquely identified by a model were correctly identified could shed light on the potential of our models as an addition to the current codes. Such evaluations are vital to assessing our models as potential annotation tools for Palga, as they would reveal insights into the accuracy and comprehensiveness of the annotations in real-world scenarios.

Third, future work should explore techniques to mitigate the decrease in performance on rare codes and complex reports (histology reports with long conclusion texts and most autopsy reports). Strategies such as data augmentation for rare codes or the development of specialized models for complex reports could be valuable. Addressing this issue is crucial because the current models tend to perform well on frequent codes and simple reports but struggle with less common codes and complex reports. By ensuring a more balanced and reliable annotation system, we can improve the overall robustness and applicability of the models across diverse datasets and clinical scenarios.

Fourth, Palga Foundation regularly adds new codes and corresponding terms to the Palga thesaurus. Whereas this poses no issue for human annotators or rule-based systems like PRAM, it presents a significant challenge for deep learning models such as the transformers investigated in this study. Our tokenizer and model weights are optimized for the codes present at training time, and newly added codes are therefore unknown to the model and cannot be generated. In addition to thesaurus updates, models are also subject to performance degradation over time due to temporal drift in clinical language and annotation conventions. This degradation is especially relevant in domains such as malignant lymphomas, where evolving terminology can limit the generalizability of models trained on older data. Future research should focus on developing practical strategies for maintaining and updating deployed models, for example through continual learning, parameter-efficient fine-tuning, retrieval-augmented generation, or dynamic vocabulary expansion, to ensure models remain aligned with current medical practice and annotation standards. In addition, LLMs with fine-tuning or cached context offer an alternative strategy for handling evolving language and imperfect supervision. Although not explored in this study, such models may provide greater flexibility and robustness in adapting to changes in clinical practice and annotation standards.

Fifth, regarding patient retrieval efficiency, it might be worth investigating the use of hierarchies in the disease codes that are used executing Palga queries. As there is no consequent hierarchy in the Palga codes, the use of other more hierarchical annotation systems like SNOMED-CT could be considered, as the SNOMED-CT codes corresponding to the Palga codes are already present in the Palga thesaurus.

## Conclusion

In this study, we investigated the performance of fine-tuned T5-based models in automatically annotating Dutch pathology reports, compared to existing manual or rule-based approaches, and whether in-domain pre-training and CD can further enhance annotation quality. We showed that our models produce good-quality annotations, particularly on histology and cytology reports with short conclusion texts. However, on histology and cytology reports and autopsy reports featuring longer and more complex conclusion texts, the models could not match the reference annotations. Additionally, we demonstrated that fine-tuning a custom pre-trained T5 model generated better annotations. Lastly, implementing a CD algorithm to enforce domain rule adherence performed on par with DD. In one of four case-based evaluations, using our models' annotations retrieved patients at the same level as the currently available annotations; in another, the Palga database codes identified only slightly more patients. In two cases, the models' annotations did not match the performance of the original codes. Interestingly, the models with the highest BLEU scores did not perform best in the case-based evaluation. This may be due to limitations in the Palga code system, which lacks a clear hierarchy, or because the models overfit to incomplete or inconsistent training annotations. Overall, our models show promise in reducing manual effort by providing machine-generated annotations for shorter reports. They may benefit from further refinement, especially for more complex cases.

## Declaration of generative AI and AI-assisted technologies in the writing process

During the preparation of this work, the author(s) used GPT-4o and GPT-o1 for grammar and spelling checks, as well as for restructuring and refining the text. After using this tool/service, the author(s) reviewed and edited the content as needed and take(s) full responsibility for the content of the publication.

## Declaration of competing interest

The authors declare that they have no known competing financial interests or personal relationships that could have appeared to influence the work reported in this article.

## Figures and Tables

**Table A.1:** Example showing difference in tokenization for “plaveiselcelcarcinoom” using both the custom tokenizer created for PaTh5.NL and the default mT5 tokenizer.

Tokenizer	Tokens
Custom PaTh5.NL tokenizer	‘_plaveiselcel’, ‘carcinoom’
Default mT5 tokenizer	‘_pla’, ‘ve’, ‘is’, ‘e’, ‘l’, ‘cel’, ‘car’, ‘cino’, ‘o’, ‘m’

**Table B.1:** Parameter ranges for investigated decoding strategies.

Strategy	Ranges
Contrastive search	Alpha: 0.7–1.0, Top_k: 1–5
Multinomial sampling	Temp: 0.5–0.7, Top_k: 20–50
Beam search	Beams: 4–9, N-gram: 1
Beam search with multinomial sampling	Beams: 4–9, N-gram: 1, Sample: True
Diverse beam search decoding	Groups: 2–3, Beams: Dynamic, Penalty: 0.1–0.5

**Table C.1:** Type statistics: Length of codes and frequency.

Type	Avg. length of codes	No. of unique codes	Avg. freq. of unique codes
T	4.64	2630	198.11
C	4.36	926	176.67
S	8.76	2757	137.59

**Table C.2:** Conclusion length group statistics.

Conclusion length group	Avg. length of codes
0–80	4.79
81–200	4.61
201–400	5.82
400+	8.67

**Table D.1:** Queries and corresponding codes. A full stop means any digit (0–9).

Query	Codes
Neuroendocrine tumors	M824…
Thyroid carcinomas	(T96… or M83491) and (M8…. or M9….)
Small intestine cancers	(T64… or T65…) and (M8…. or M9….)
Colorectal carcinomas	(T67… or T68…) and (M8…. or M9….)

**Table E.1:** Case-based evaluation results for four entities: neuroendocrine tumors, thyroid carcinomas, small intestine cancers, and colorectal carcinomas. For each model, the number of reports retrieved by the original codes (*O*), by the model (*M*), only by the original codes (*O*\*M*), only by the model (*M\O*), and by both (*O*∩*M*) are shown.

Model type	*O*	*M*	*O*\*M*	*M*\*O*	*O*∩*M*
*Neuroendocrine tumors*					
mT5	4962	4440	975	453	3987
PaTh5.NL-DD	4962	3727	1531	296	3431
PaTh5.NL-CD	4962	3367	1888	293	3074
PRAM	4962	4249	1235	522	3727

*Thyroid carcinomas*					
mT5	1757	1664	213	120	1544
PaTh5.NL-DD	1757	1470	363	76	1394
PaTh5.NL-CD	1757	1539	295	77	1462
PRAM	1757	1641	247	131	1510

*Small intestine cancers*					
mT5	5823	5138	1020	335	4803
PaTh5.NL-DD	5823	4836	1284	297	4539
PaTh5.NL-CD	5823	4752	1346	275	4477
PRAM	5823	5703	751	631	5072

*Colorectal carcinomas*					
mT5	6698	6673	348	323	6350
PaTh5.NL-DD	6698	6699	340	341	6358
PaTh5.NL-CD	6698	6366	582	250	6116
PRAM	6698	5866	1144	312	5554

**Table F.1:** Evaluation metrics for different models.

Model	BLEU	ROUGE	WER	CER	MER	WIL	WIP
mT5	30.46	60.85	51.25	39.57	46.43	60.47	39.53
PaTh5.NL-DD	34.65	61.68	54.76	42.04	46.62	60.76	39.24
PaTh5.NL-CD	33.50	60.84	55.67	43.09	47.68	61.88	38.12
PRAM	9.95	36.52	146.86	125.81	73.47	85.13	14.87

**Table F.2:** Correlation of evaluation metrics with BLEU score.

Metric	Correlation with BLEU
ROUGE	+0.991
WER	−0.982
CER	−0.983
MER	−0.985
WIL	−0.984
WIP	+0.984

## Appendix A Tokenization

As part of the pre-training process, we trained a custom tokenizer to ensure optimal alignment between the input text and the available tokens compared to the default tokenizer. Our custom tokenizer, identical to the original T5 implementation, was implemented using the WordPiece algorithm with a vocabulary size of 32,128.^48^ The WordPiece algorithm breaks down unknown words into smaller sub-words, called sub-tokens, that it does know. The custom tokenizer was trained using HuggingFace's train_from_iterator function.^12^

Before finding the most optimal sub-tokens using this function, we first added all 6296 unique terms in the Palga thesaurus to the tokenizer. This ensured that the tokenizer tokenized these words as a single token.

To measure the performance of this custom tokenizer, we compared it to the default tokenizer used for fine-tuning our mT5 model. On average, the custom tokenizer required 5.05 tokens to tokenize the reference annotations of our pre-training data, a 65% reduction compared to the 14.48 tokens required by the mT5 tokenizer. Additionally, to tokenize the input text, the custom tokenizer required an average of 27.07 tokens, a 46% reduction compared to the 50.3 tokens required by the default mT5 tokenizer.

An example of this difference can be seen when tokenizing the Palga code P11400 (biopt). The mT5 tokenizer splits the code into three tokens (P, ##11, ##400), whereas the custom PaTh5.NL tokenizer treats the entire code as a single token (P11400). This example highlights the tokenizer's improved handling of task-specific terms. Another comparison for clinical text is shown in Table A.1, where “plaveiselcelcarcinoom” is tokenized by both the custom and default tokenizers.

## Appendix B Decoding strategies hyperparameters

Table B.1 displays the decoding strategies, hyperparameters, and the range of values investigated for the parameter.

## Appendix C Conclusion text complexity

Generally, annotations for reports with more complex conclusion texts are more challenging for our models to generate accurately. We assess annotation complexity using three key statistics:

1. Average length of the annotation (in number of codes): Longer annotations have more codes, which increases the risk of missing codes or predicting incorrect ones.
2. Number of unique codes used: The diversity of codes used within the annotation is crucial because a greater variety of codes suggests higher complexity, making accurate predictions more challenging.
3. Average frequency of each unique code used: The performance of our models tends to decline as the frequency of a code decreases. Infrequent codes are harder to predict correctly, as indicated by the proportional decrease in model performance in Fig. 3.

As shown in Table C.1, autopsy conclusion texts (Type S) exhibit a higher level of complexity compared to types C and T. This complexity is reflected in several key metrics: Type S has the longest average annotation length, the greatest number of unique codes, and a relatively lower average frequency of these codes. These factors indicate that Type S annotations are more challenging for our models to annotate accurately.

Additionally, Table C.2 illustrates that annotation complexity tends to rise with the length of the conclusion. Annotations in the 400+ length group have the highest average length of codes, indicating greater complexity.

## Appendix E Case-based evaluation: Report counts

Table E.1 shows the report counts on which the patient counts in Table 6 are based. The table presented here is a mirror image of Table 6, with retrieved report counts instead of patient counts. As before, *O* is the total number of reports retrieved by the original codes, *M* is the total retrieved by the model, *O*\*M* are reports retrieved only by the original codes, *M*\*O* are reports retrieved only by the model, *O*∩*M* are those retrieved by both. Whereas the relative performance of each model is the same as for patient retrieval, for colorectal carcinomas the results differ. Here, the PaTh5.NL-DD model identified the highest number of reports (*M* = 6699), followed closely by mT5 (*M* =6673) and PaTh5.NL-CD (*M* =6366), with PRAM identifying the fewest reports (*M* =5866). Whereas all four models identified a similar number of reports that were missed by the original codes (*M*\*O*), PRAM had significantly fewer reports in its intersection with the original codes (*O*∩*M* = 5544) compared to mT5 (6350), PaTh5.NL-DD (6358), and PaTh5.NL-CD (6116). Remarkably, the differences in the number of retrieved reports are not reflected in the number of patients retrieved in this category, with the number of patients retrieved being almost equal for all models (mT5 = PaTh5.NL-DD=PaTh5.NL-CD: 1484; PRAM: 1481). Also, there was no contribution by the models to the number of patients retrieved beyond those identified by the original codes.

## Appendix F Additional metrics in quantitative evaluation

Table F.1 shows additional metrics to the BLEU scores we calculated: WER, CER, MER, WIL, WIP, and ROUGE. These were calculated on the results of Experiment 1 (the whole FT test dataset).

The relationship (correlation coefficient) between BLEU and the alternative metrics is shown in Table F.2.

## Footnotes

3 https://www.palga.nl.

4 Reports of these types were excluded because their annotations are in a standard, protocol-generated format, and never created manually.

5 Available from https://huggingface.co/google/mt5-small.

6 Nederlandse Kankerregistratie, https://iknl.nl/nkr, Integraal kankercentrum Nederland, https://iknl.nl.

7 Available from https://graphviz.readthedocs.io/en/stable/index.html.

8 Available from https://matplotlib.org/.

9 Available from https://github.com/mjpost/sacrebleu.

10 Available from https://www.python.org/.

11 See Appendix D.
